# Notes and Notices

**Published:** 1883-07

**Authors:** 


### NOTES AND NOTICES.

REMOVALS.—Dr. C. G. Wilson, from St. Clair, Michigan, to St. Cloud, Minnesota; Dr. S. A. Kimball, from Boston to Melrose, Massachusetts; Dr. A. F. Randall, from Lexington, Michigan, to Brodhead, Wisconsin; Dr. J. W. Thomson, from New Haven to No. 132 East Fiftieth Street, New York city; Dr. J. H. Way will be at "The Aldine," Atlantic City, for the summer.

PROFESSOR FRANKLIN has resigned the chair of surgery in the homœopathic department at Ann Arbor and will practice in St. Louis.

A NEW JOURNAL, to be called *The Homœopathic Leader*, will soon be started in New York under the editorship of Dr. Walter Y. Cowl, with distinguished associate editors for the various specialties. We wish this new journal all success, and hope it may prove to be a leader of *true* Homœopathy. We have now a dozen or more journals, leaders of *false* Homœopathy.

THE ALLOPATHIC "STAR CHAMBER."—The American Medical Association decides all questions of faith for the orthodox regular, and is itself ruled over by a "Star Chamber" known as the "Judicial Council." So this "Judicial Council" rules the sixty thousand regulars of America and is to be responsible to no one! At the last meeting of the American Medical Association this "Judicial Council," to prevent any "new coders" from entering as members of the meeting, wisely decided to allow no one to register, as member or delegate, until he signed a pledge professing his allegiance to the old code and his abhorrence of the new! This, of course, made the Convention unanimous in favor of the old code! A packed convention!! One of the delegates signed this pledge under protest, and *was expelled from the Association for so protesting!* The New York *Times* comments thusly upon his expulsion:

"The labors of the American Medical Association were crowned at its final session by the expulsion of Dr. Goodwillie, of this city, upon the express ground that he adhered to the Code of Ethics of the New York State Society. In other words, a physician whose standing is not challenged in any other respect is declared unworthy of professional association because he reserves the right to attend a patient in consultation with an 'irregular' physician of the patient's own choosing, whenever, in his judgment, any emergency requires him to do so. And this reservation is regarded as the violation of a code of 'ethics'! The doctors who take this view would appear to be cruel bigots if they did not present so much more prominently the aspect of simple geese. This is not the spirit of a learned profession; it is the spirit of an ignorant trades-union, bent upon punishing 'rats.' The men who made it and who adhere to it must believe not so much that it is the business of physicians to heal the sick as that it is the business of the sick to furnish constant and remunerative employment to a carefully limited number of 'regular' physicians."

The American Institute should get up such a Star Chamber and allow no one to register as a member who does not alternate his remedies and use crude doses, apply blisters, etc. Such a Star Chamber would soon settle the dose and other vexed questions.

THE AMERICAN INSTITUTE.—It is said about three hundred were in attendance at the Niagara meeting. The following officers were chosen: Professor J. C. Saunders, of Cleveland, President; Professor T. F. Allen, New York, Vice-President; Dr. J. C. Burgher, Pittsburg, General Secretary; E. M. Kellogg, New York city, Treasurer. An Inter-Collegiate Committee was established, consisting of two delegates from each of the eleven homœopathic colleges.

